# Status of Fungicide Resistance and Physiological Characterization of Tebuconazole Resistance in *Rhizocotonia solani* in Sichuan Province, China

**DOI:** 10.3390/cimb44100330

**Published:** 2022-10-13

**Authors:** Changwei Gong, Min Liu, Dan Liu, Qiulin Wang, Ali Hasnain, Xiaoxu Zhan, Jian Pu, Yueyang Liang, Xuemei Liu, Xuegui Wang

**Affiliations:** 1 College of Agriculture, Sichuan Agricultural University, Chengdu 611130, China; 2 Rice Institute, Sichuan Agricultural University, Chengdu 611130, China; 3 State Key Laboratory of Crop Gene Exploration and Utilization in Southwest China, Sichuan Agricultural University, Chengdu 611130, China

**Keywords:** rice sheath blight, tebuconazole, epoxiconazole, CYP51 mutation, binding energy

## Abstract

The resistance prevalence of chemical fungicides has caused increasingly serious agro-ecological environmental problems. However, there are few previous reports about resistance to succinate dehydrogenase (SDHI) or sterol demethylation inhibitor (DMI) in *Rhizoctonia solani*, one of the main agro-diseases. In this study, the fungicide resistance of 122 *R. solani* isolates in Sichuan Province was monitored by the mycelial growth rate method. Results showed that all isolates were susceptible to hexaconazole and most isolates were susceptible to thifluzamide, except for the field isolate MSRS-2-7 due to a moderate resistance to thifluzamide (16.43-fold resistance ratio, RR), compared to the sensitivity baseline of thifluzamide (0.042 μg/mL EC_50_ values). On the contrary, many isolates showed moderate or high resistance to tebuconazole (10.59- to 60.78-fold RR), reaching EC_50_ values of 0.54~3.10 μg/mL, especially for a highly resistant isolate LZHJ-1-8 displaying moderate resistance to epoxiconazole (35.40-fold RR due to a 3.54 μg/mL EC_50_ value). The fitness determination found that the tebuconazole-resistant isolates showed higher fitness cost with these characteristics, including a lower growth rate, higher relative electric conductivity, an increased ability to tolerate tebuconazole, and high osmotic pressure. Four new mutations of cytochrome P450 sterol 14α-demethylase (CYP51), namely, S94A, N406S, H793R, and L750P, which is the target for DMI fungicides, was found in the tebuconazole-resistant isolates. Furthermore, the lowest binding energy with tebuconazole was also found in the LZHJ-1-8 isolate possessing all the mutations through analyses with Discovery Studio software. Therefore, these new mutation sites of CYP51 may be linked to the resistance against tebuconazole, and its application for controlling *R. solani* should be restricted in some areas.

## 1. Introduction

Rice sheath blight, caused by *Rhizoctonia solani* Kuhn AG1-1A [teleomorph *Thanatephorus cucumeris* (A. B. Frank) Donk], is a common disease in rice and mostly occurs under high temperature and humidity. The disease can produce incomplete grains, more scum, and even lodging [1]. It can cause a yield loss of 20–50% in susceptible rice cultivation areas and shows an increasing trend due to the irrational use of nitrogen fertilizer and change in global climate conditions [2]. The measures to control *R. solani* mainly include strengthening management of field fertilizer and water, reduction in bacterial sources, and cultivation of resistant varieties [3]. However, the application of chemical fungicides is the most popular and effective control measure [4]. In rice production, the common chemical fungicides for controlling rice sheath blight consist of succinate dehydrogenase inhibitors (SDHIs) or sterol demethylation inhibitors (DMIs), such as thifluzamide, hexaconazole, tebuconazole, and epoxiconazole [5,6].

However, as a result of their increased usage, the susceptibility of pathogens to these fungicides is decreasing, and even fungicide-resistant isolates have emerged, leading to significant difficulties for controlling these diseases. The resistance towards some quinone-outside inhibitor (QoI) fungicides has been found in *R. solani* to affect rice and soybean in the southern United States. Ajayi-Oyetunde et al. [4] reported that resistance against SDHI and DMI classes of fungicides was not identified for *R. solani*. Chen et al. [7] and Mu et al. [8] determined the sensitivity of *R. solani* isolates towards thifluzamide in China, and found that all isolates were extremely susceptible to thifluzamide, with an average EC_50_ value of 0.05 or 0.0351 μg/mL; while Mu et al. [8] obtained nine thifluzamide-resistant isolates using thifluzamide-amended medium or UV radiation. Ajayi-Oyetunde et al. [9] found that all isolates of *R. solani* were susceptible to both SDHI and DMI fungicides, and more susceptible against SDHI. Two years later, Suemoto et al. [10] reported that DMI fungicides were losing their efficacy against *Zymoseptoria tritici* and *Pyrenophora teres* in the regions of west Europe, where these cereals were intensively produced. Although there are relatively few available reports about *R. solani* resistance to DMI, its resistance for other diseases have been identified, such as *Cercospora beticola* [11], *Monilinia fructicola* [12], and *Colletotrichum gloeosporioides* [13].

DMIs are considered as fungicides for controlling *R. solani*, as they inhibit cytochrome P450 sterol 14α-demethylase (*CYP51*), and thus interfere with the biosynthesis of ergosterol, a primary sterol in fungal membranes [14]. DMI resistance is generally considered to be associated with point mutations or overexpression of CYP51 and efflux pump overexpression. Sun et al. [15] found that a new putative sterol in PdCYP51B was involved in resistance to imazalil and other DMI fungicides; Cools et al. [16] found that *MgCYP51* overexpression in *Mycosphaerella graminicola* isolates conferred a novel azole fungicide sensitivity; Wei et al. [13] found that not only overexpression, but also mutations, conferred DMI-resistance in *C. gloeosporioides*. Other resistance mechanisms include the increased expression of ATP-binding cassette (ABC) transporters and major facilitator superfamily (MFS) transporters encoding efflux pumps [17].

To the best of our knowledge, thifluzamide, hexaconazole, tebuconazole, epoxiconazole, and their compound fungicides are widely used in the control of *R. solani* in Sichuan Province. However, the sensitivity of *R. solani* to these four fungicides and the potential resistance mechanism in *R. solani* to tebuconazole have been little studied. In this paper, the resistance levels of *R. solani* isolates, collected from the different rice-cultivating districts, against hexaconazole, tebuconazole, epoxiconazole, and thifluzamide were determined; furthermore, their fitness and the resistance mechanism were also analyzed to provide a theoretical basis for formulating integrated pest management for *R. solani*.

## 2. Materials and Methods

### 2.1. Isolation of R. solani

Rice leaves or stems with typical symptoms of *R. solani* infection were collected in 2018–2019 from different rice cultivation regions, namely, Chengdu Chongzhou (CDCZ), Chengdu Dayi (CDDY), Chengdu Pixian (CDPX), Meishan Renshou (MSRS), Zigong Rongxian (ZGRX), Luzhou Hejiang (LZHJ), and Neijiang Longchang (NJLC) in Sichuan Province, China (Table S1). The *R. solani* isolates were separated on the water agar medium following the description of Chen et al. [7] with some modifications. After the infected rice stems were cut into 6 mm^2^ pieces, they were disinfected in 0.5% (*v/v*) sodium hypochlorite for 1 min and 75% (*v/v*) ethanol for 30 s, rinsed three times with sterile water, and cultured on the water agar medium having streptomycin and 1% lactic acid at 28 °C under the darkness condition. After two days, a total of 122 *R. solani* isolates was separated from the edge of mycelia, and then transferred to the PSA medium. All isolates were further identified by mycelia morphology and amplification with primer pairs ITS1/ITS4 [18].

### 2.2. Fungicides and Chemicals

Four technical fungicides, namely, thifluzamide (95% available ingredient (a. i.) Zhejiang Yulong Biotechnology Co., Ltd., Jiaxing, China), hexaconazole (95.38% a. i. Yancheng Yuenong chemical Co., Ltd., Yancheng, China), tebuconazole (95% a. i. Zhejiang Hangzhou Yulong chemical Co., Ltd., Hangzhou, China), and epoxiconazole (97% a. i. Jiangsu Huifeng Biological Agriculture Co., Ltd., Yancheng, China), were dissolved in analytical grade acetone (>99.5%) to prepare 1000 μg/mL stock solutions. The physiological biochemical reagents, including glucose and sodium chloride (NaCl), were purchased from Chengdu Kelong Chemical Reagent Co., Ltd. (Chengdu, China). The drug-containing medium was made by mixing 1 mL fungicide stock solution diluted by 0.1% Tween-80 and 9 mL PSA medium; 1 mL 0.1% Tween-80 consisting of an equal volume of acetone and 9 mL PSA medium was used as the blank control (drug-free medium).

### 2.3. Determination of Resistance Frequency of R. solani

The minimum inhibitory concentration (MIC) was used to determine the resistance frequency of *R. solani* [19]. The MICs of four fungicides were based on their lowest inhibitory concentration [20] against an indoor susceptible isolate obtained from Southwest Crop Genetic Resource Discovery and Utilization Laboratory of Sichuan Agricultural University; the MICs of thifluzamide, hexaconazole, tebuconazole, and epoxiconazole were 5, 15, 20, and 10 μg/mL, respectively. The 122 isolates were placed on a PSA medium plate, previously stored in a refrigerator at 4 °C, and activated at a constant temperature of 28 °C for 36 h; then, mycelia with a 5 mm diameter were punched out with a hole punch, and inoculated on PSA medium containing the identified MICs. After culturing in the dark at 28 °C for 36 h, those isolates that could not grow normally were identified as susceptible isolates; on the contrary, the isolates that could grow normally were identified as resistant isolates, and the mycelia diameter was measured by the cross method [21]. The mycelia diameter grown on the drug-containing medium was marked A, and that on the drug-free medium was marked B. The occurrence frequency of susceptible and resistant isolates, and the mycelial growth inhibition rate, were calculated [22]. Mycelial growth inhibition rate (%) = [1 − (A − 5 mm)/(B − 5 mm)] × 100%

### 2.4. Sensitivity Baseline or Resistance Ratio of R. solani to the Tested Fungicides

According to the resistance frequency and mycelial growth inhibition rate results, some representatives from sensitive and resistant (with a least inhibition rate) isolates of *R. solani* to thifluzamide, hexaconazole, tebuconazole, and epoxiconazole were selected, and their EC_50_ values against these four fungicides were determined by the mycelial growth rate method [23]. Each treatment was replicated three times. A mycelium with a diameter of 5 mm was inoculated into the center of the drug-containing medium, and the drug-free medium was used as a blank control. After culturing at 28 °C for 36 h, the mycelia diameter was measured by the cross method [21]. A regression equation was derived by correlating the log10 of inhibitor concentration and the probability value of the mycelial growth inhibition rate, while effective concentration for 50% inhibition rate (EC_50_) was calculated from the regression equation [7]. Resistance ratio (RR) was obtained as the ratio of EC_50_ value for resistant isolates to EC_50_ value for sensitive isolates.

### 2.5. Fitness Determination of Tebuconazole-Resistant Isolates

Referring to the method reported by Dolores et al. [24], to test the osmotic sensitivity of the tebuconazole-sensitive or resistant isolates to glucose, mycelium plugs with 5 mm diameter were punched at the edge of the mycelia, and inoculated onto PDA medium containing 1%, 2%, 4%, and 8% glucose, after activation at 28 °C for 36 h. Then, the mycelia growth diameter was measured by the cross method, and each treatment was repeated 3 times.

To test the osmotic sensitivity of these isolates towards NaCl, mycelium plugs with a 5 mm diameter from the edge of 36 h activated mycelia were transferred into PSA medium containing mass concentration of 0, 1.25, 2.5, 5, 10, 20, 40, and 80 g/L NaCl. Each isolate was incubated at 28 °C for 36 h with three replicates. The growth diameters of mycelia on medium with different concentrations of NaCl were measured by the cross method [21].

### 2.6. Determination of Cell Membrane Permeability

The cell membrane relative permeability rate for tebuconazole-sensitive or resistant isolates was evaluated according to the described method [25] with some modifications. The activated mycelia were respectively inserted into the PDB medium, and shaken (120 r/min) for 3 d; then, the fresh mycelium was collected, and later washed with double-distilled sterile water and vacuum filtered. Then, 0.5 g of the fresh weight was put into a conical triangular flask, consisting of 0, 0.5, 1.0, 5.0, and 25.0 µg/mL tebuconazole diluted with double distilled sterile water. After shaking (120 r/min) in a constant temperature water bath at 28 °C for 0, 5, 10, 15, 30, 60, 90, 120, 180, 240, 300, 360, 420, and 540 min, the conductivity was measured using a DDS-11A meter, and the boiled dead mycelium was treated as a control. Each treatment was repeated 3 times.

### 2.7. Clone of CYP51 Gene from R. solani Isolates

Genomic DNA from mycelium of *R. solani* isolates were extracted using the EasyPure Plant Genomic DNA Kit (TransGen Biotech, Beijing, China) according to the manufacturer’s recommendations [26]. The PCR primers (Table S2), based on the genome of *R. solani*, were used to amplify the CYP51 gene fragment of the tebuconazole-sensitive or resistant isolates. I-5TM 2 × High Fidelity Master Mix DNA Polymerase (Molecular Cloning Laboratories, Beijing, China) was used in the PCR. PCR was conducted with a PCR cycle of 98 °C for 3 min, 39 cycles of 98 °C for 10 s, 55 °C for 15 s, and 72 °C for 20 s, ending with an extension at 72 °C for 5 min. PCR products were sequenced (Qingke Biotechnology Co., Ltd., Beijing, China), and the gene sequences of the isolates were measured and analyzed with ClustalX2 software, while the alignment results were visualized with ESPript 3.x software (https://espript.ibcp.fr/ESPript/cgi-bin/ESPript.cgi, accessed on 7 September 2022).

### 2.8. Functional Domain and Structural Analysis of CYP51 Gene

The protein sequences of related fungal CYP51 were downloaded from the NCBI (https://www.ncbi.nlm.nih.gov/, accessed on 7 September 2022), the conservative functional domains of CYP51 were retrieved by the motif-search (https://www.genome.jp/tools/motif/, accessed on 7 September 2022) and MEME (https://meme-suite.org/meme/tools/meme, accessed on 7 September 2022), while the evolutionary tree was constructed with MEGA7.0 software by the maximum likelihood (ML) method [27]. The results were visualized with TBtools software.

Three-dimensional structural modeling of CYP51 from tebuconazole-sensitive isolate was performed by the I-TASSER (http://zhanglab.ccmb.med.umich.edu/I-TASSER, accessed on 7 September 2022), while the three-dimensional structure model of different mutants was built though the Swiss-Model software (https://swissmodel.expasy.org/, accessed on 7 September 2022) with CYP51 model of tebuconazole-sensitive isolate as the template; the tebuconazole structure was downloaded from the PubChem. The binding model and affinity between tebuconazole and the different CYP51 proteins was evaluated using Discovery Studio [28].

### 2.9. Statistical Analysis

The EC50 values for each isolate, cell membrane relative permeability rate, and mycelia growth diameter were compared using analysis of variance (ANOVA) followed by Student’s *t*-test for multiple comparisons (*p* < 0.05) with the SPSS version 17.0 software package (IBM).

## 3. Results

### 3.1. Resistance Frequency of R. solani in Sichuan Province

**Figure 1:**
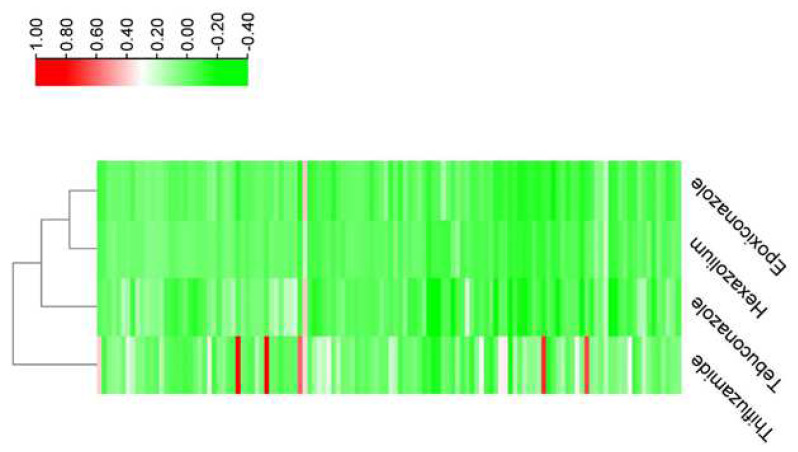
The relative inhibition rate of different isolates to different fungicides, relative to the indoor susceptible isolate obtained from Southwest Crop Genetic Resource Discovery and Utilization Laboratory of Sichuan Agricultural University.

The results of resistance frequency for all 122 *R. solani* isolates showed that the resistance frequency to thifluzamide, hexaconazole, tebuconazole, and epoxiconazole was 65.57%, 45.08%, 47.54%, and 36.07%, respectively (Table 1). The resistance frequency of *R. solani* in different areas was also different, and the resistance frequency of hexaconazole reached up to 100% in Chengdu Dayi (Table 1). According to the inhibition rate of different isolates to different fungicides, the relationship between tebuconazole and epoxiconazole was higher than that with thifluzamide (Figure 1).

### 3.2. Sensitivity Baseline of R. solani to Four Test Fungicides

The measurement results of sensitivity to these four test fungicides of 10 representative isolates indicated that the EC_50_ values to thifluzamide ranged from 0.03 to 0.05 μg/mL, and the average EC_50_ value was 0.042 μg/mL, among which the EC_50_ values of MSRS-2-17, ZGRX-2-19, CDDY1-2, and ZGRX-3-1 were 0.05 μg/mL, those of CDPX-1-7, ZGRX-2-13, MSRS-2-3, and ZGRX-1-3 were 0.04 μg/mL, and those of the rest representative isolates were 0.03 μg/mL. The range of EC_50_ values to hexaconazole was from 0.02 to 0.07 μg/mL, their average EC_50_ value reached 0.051 μg/mL, and the EC_50_ values of four representative isolates were 0.06 μg/mL; the most susceptible isolate was MSRS-3-13 with a 0.02 μg/mL EC_50_. The scope of the EC_50_ values to tebuconazole was 0.02–0.23 μg/mL, possessing an average EC_50_ of 0.109 μg/mL, among which the least susceptible isolate was MSRS-2-13, achieving 11.5-fold that of MSRS-3-16 (0.02 μg/mL EC_50_). The range of EC_50_ to epoxiconazole was 0.03–0.15 μg/mL, and the average EC_50_ value was 0.100 μg/mL, among which the least susceptible isolate was 5.0 times the most susceptible isolate (Table 2).

### 3.3. Resistance Levels of R. solani Isolates to Four Fungicides

The results of resistance levels of four fungicides showed that the EC_50_ values of *R. solani* isolates against thifluzamide were distributed between 0.15 and 0.69 μg/mL, and their resistance ratio (RR) reached 1.43–16.43-fold compared with its sensitive baseline. Most reached sensitive or low resistant levels, apart from MSRS-2-7 with a moderate resistance to thifluzamide isolates (16.43-fold RR); and based on the sensitive baseline of hexaconazole, all isolates showed sensitivity levels with RR values of 2.0- to 4.5-fold, whose EC_50_ values ranged from 0.12 to 0.18 μg/mL. According to the sensitive baseline of tebuconazole, whereas many isolates having moderate or high-level resistance were observed (10.59- to 60.78-fold RR), and their EC_50_ values ranged from 0.54 and 3.1 μg/mL, in which the highest resistance levels were for MSRS-1-3 and LZHJ-1-8, reaching 52.16- and 60.78-fold, respectively. Furthermore, compared to the sensitivity baseline of epoxiconazole, the RR values of the representative resistant isolates were 1.90- to 35.40-fold, and their EC_50_ values were distributed in the range 0.14–3.54 μg/mL. Most retained a sensitive level, in addition to the moderate resistance LZHJ-1-8, which also displayed high level resistance to tebuconazole, of 35.40-fold (Table 3).

### 3.4. Fitness of Tebuconazole-Resistant Isolates

**Figure 2:**
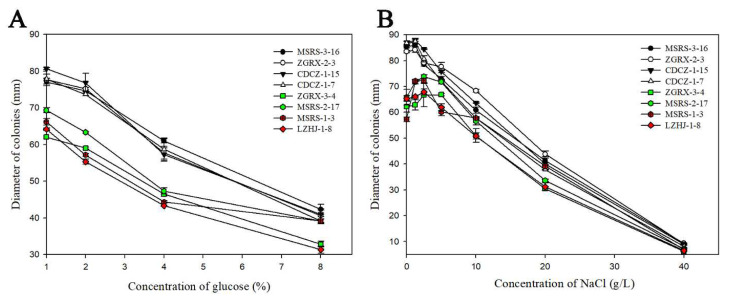
Fitness determination of tebuconazole-susceptible and -resistant isolates on PDA medium with different concentrations of glucose (**A**) or NaCl (**B**). Red or green indicates high or medium resistance to tebuconazole, respectively; black or white indicates tebuconazole-susceptible isolates. The F_7,23_ values of the colony diameters of the different isolates on the PDA with 1% glucose, 2% glucose, 4% glucose, and 8% glucose were 114.368, 11.767, 1.072, and 32.483; the *p* values were = 0.000 < 0.0001, = 0.000 < 0.01, = 0.426 > 0.05, and = 0.000 < 0.01, respectively. The F_7,23_ values of the colony diameters of the different isolates on the PDA with 0, 1.25, 2.5, 5, 10, 20, 40, and 80 g/L NaCl were 83.665, 110.603, 12.916, 37.355, 23.083, 36.933, and 15.752, respectively; the *p* values all were = 0.000 < 0.0001.

On PDA medium with the different concentrations of glucose, the mycelia growth trend of tebuconazole-sensitive and resistant isolates was basically same, and their mycelia diameters decreased with the increase in glucose concentration. The mycelia growth diameters of tebuconazole-resistant isolates (31–69 mm) were always smaller than those of the tebuconazole-sensitive isolates (39–80 mm). However, when the glucose concentration was increased from 4% to 8%, the decreased magnitudes of the tebuconazole-resistant isolates ZGRX-3-4, MSRS-2-17, MSRS-1-3, and LZHJ-1-8 (13.67, 8.17, 5.17, and 12.00 mm, respectively) were significantly lower than those of tebuconazole-sensitive isolates MSRS-3-16, ZGRX-2-3, CDCZ-1-15, and CDCZ-1-7 (18.67, 17.08, 16.25, and 19.50 mm, respectively), which indicates that the tebuconazole-sensitive isolates were more sensitive to osmotic stress than those in the tebuconazole-resistant isolates (Figure 2A).

At low concentrations of NaCl (0~1.25 g/L), all the mycelia growth diameters of the tebuconazole-sensitive (84–88 mm) and -resistant isolates (57–72 mm) improved with the increase in NaCl concentration; and within the concentration range of 1.25–5 g/L, the mycelia growth diameter of tebuconazole-sensitive isolates (72–85 mm) decreased. This was not consistent with those of tebuconazole-resistant isolates, for which the mycelia growth diameter was increased from 62 to 74 mm. At high concentrations of NaCl that exceeded 5 g/L, all the mycelia growth diameters of tebuconazole-sensitive (8–68 mm) and -resistant isolates (6–58 mm) decreased with the increase in NaCl concentration; until the NaCl concentration was more than 40 g/L, all the isolates could not grow up (Figure 2B). Although mycelia growth diameters of tebuconazole-resistant isolates were always smaller than those of the tebuconazole-sensitive isolates when the NaCl concentration exceeded 1.25 g/L, the reduction magnitudes of the tebuconazole-resistant isolates (57.83, 66.83, 67.17, and 61.00 mm, respectively) were lower than those of tebuconazole-sensitive isolates (81.00, 79.17, 83.00, and 82.33 mm, respectively), and their difference was extremely significant (*p* = 0.000 < 0.01). This indicates that the tebuconazole-sensitive isolates were more sensitive to osmotic stress (Figure 2B).

### 3.5. Cell Membrane Permeability

**Figure 3:**
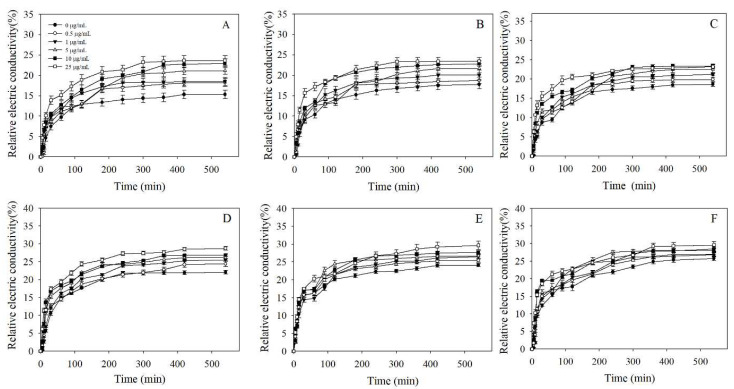
The relative electric conductivity of tebuconazole-resistant and -susceptible isolates exposed to varying concentrations of tebuconazole. Bars denote the standard deviation of two experiments: (**A**–**C**) represent tebuconazole-susceptible isolates MSRS-3-16, ZGRX-2-3, and CDCZ-1-15; (**D**–**F**) represent tebuconazole-resistant isolates ZGRX-3-4, MXRS-1-3, and LZHJ-1-8.

The relative electric conductivity of all isolates gradually increased with the promoted concentration of tebuconazole (0, 0.5, 1.0, 5.0, 25.0 μg/mL) and treatment time duration. In addition, the relative electric conductivity of all isolates increased significantly within 60 min, and tended to be stable after 240 min. Even though the relative electric conductivity values of tebuconazole-resistant isolates ZGRX-3-4, MXRS-1-3, and LZHJ-1-8 (21.32–29.48%) were generally higher than those of tebuconazole-sensitive isolates (16.62–23.46%) after 240 min of the exposure time under different concentrations of tebuconazole, the change values of relative conductivity of highly tebuconazole-resistant isolates MXRS-1-3 (24.12% to 29.25%) and LZHJ-1-8 (25.49% to 29.48%) were smaller than those of tebuconazole-sensitive isolates MSRS-3-16 (15.13% to 23.40%) and ZGRX-2-3 (17.15% to 23.46%); in particular, LZHJ-1-8 showed a minimal change (3.99%) when the concentration of tebuconazole increased from 0 to 25 μg/mL (Figure 3).

### 3.6. Functional Domain Analysis of Sterol 14α-Demethylase (CYP51)

**Figure 4:**
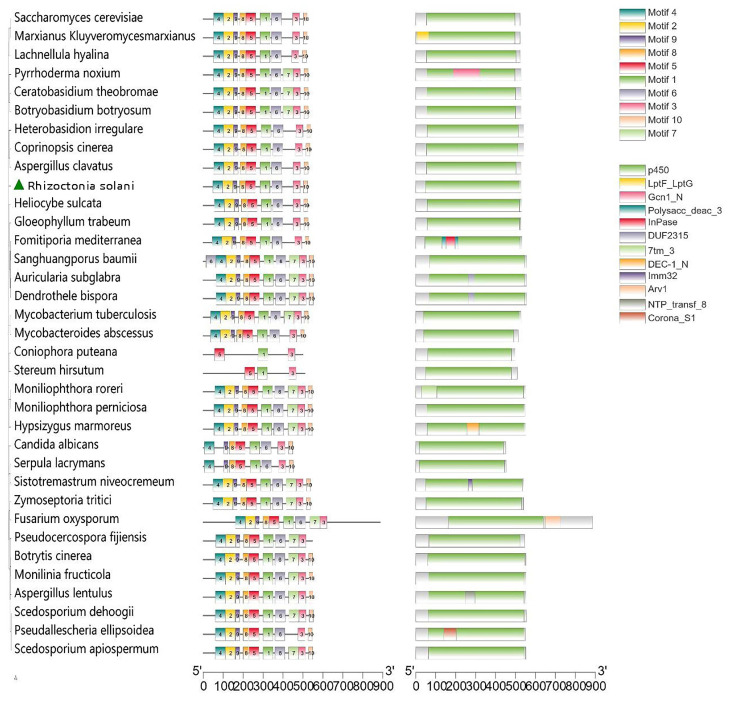
The evolutionary tree and gene structure of sterol 14α-demethylase from different species.

The CYP51 of all selected species, except for *Coniophora puteana* and *Stereum hirsutum*, contained motif5, motif1, and motif6 domains in series using the Meme-search. Furthermore, there was no other domain between motif5 and motif1, which were composed of 50 amino acid residues; motif6 was composed of 36 amino acid residues and possessed absolutely conserved amino acid residues (EXLR, a helix K motif); and the heme-binding signature motif (PFxxGxxxCxG) was located in motif3 which was behind motif6 (Figure 4 and Figure S1). According to the evolutionary tree and gene structure diagram, *R. solani* CYP51 had high homology with *Heliocybe sulcate*, *Gloeophyllum trabeum*, *Fomitiporia mediterranea*, and *Sanghuangporus baumii*, showing a similar gene structure (Figure 4).

### 3.7. Detection of CYP51 Mutation of Tebuconazole-Susceptible and -Resistant Isolates

**Figure 5:**
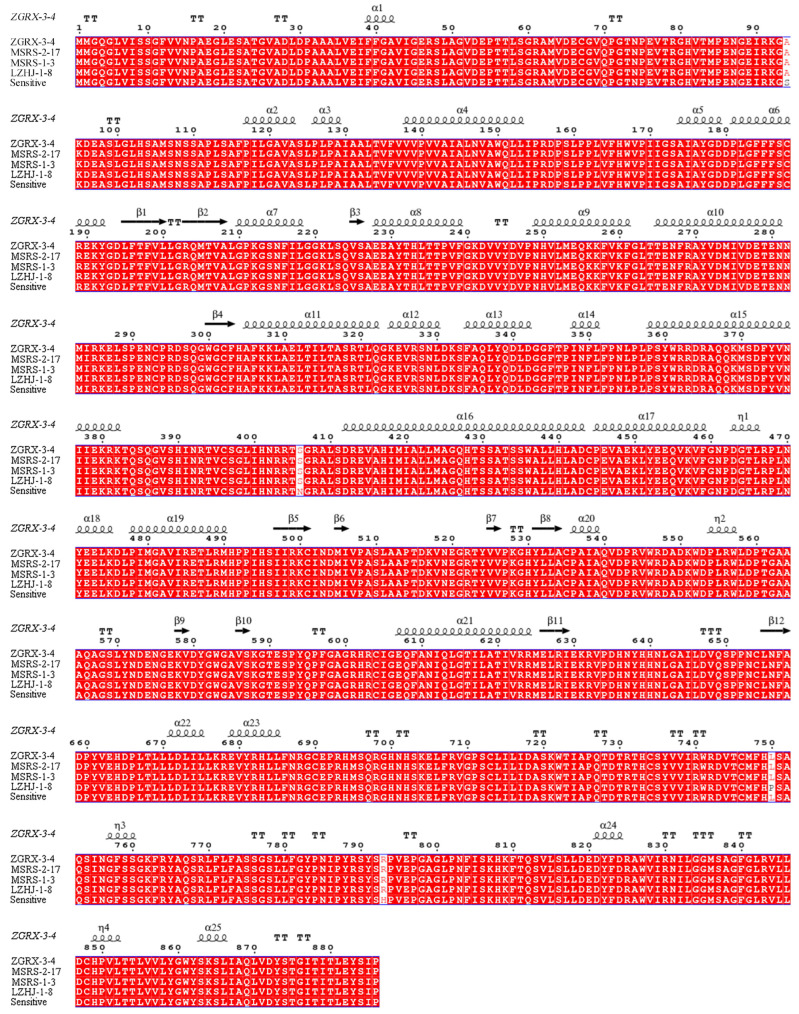
Sequence alignment of amino acid of CYP51 in different isolates.

After sequencing, there were three resistant isolates (ZGRX-3-4, MSRS-2-17, MSRS-1-3) which had three mutated positions, S94A, N406S, and H793R, respectively. In another resistant isolate LZHJ-1-8, apart from S (serine) to A (alanine) at position 94, N (aspartic acid) to S (serine) at position 406, L (leucine) to P (proline) at position 750, and another mutation, H (histidine) to R (arginine) at position 793 were identified (Table 4 and Figure 5). All mutations of S94A, N406S, L750P, and H793R were in the irregular coils of the CYP51 protein (Figure 5).

### 3.8. Effects of Mutations on the Affinity of CYP51 Protein and Tebuconazole

**Figure 6:**
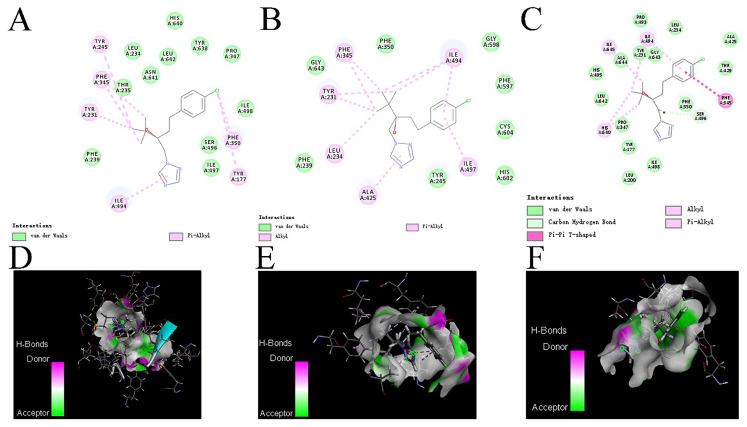
The interaction models for the CYP51-tebuconazole complex: (**A**,**D**) the interaction models of CYP51 and tebuconazole in the susceptible isolates, respectively; (**B**,**E**) those of CYP51^S94A,N406S,H793R^ and tebuconazole, respectively; (**C**,**F**) those of CYP51 ^S94A,N406S,L750P,H793R^ and tebuconazole, respectively.

In the interaction models of CYP51 and tebuconazole (Figure 6), the binding energy of CYP51 and tebuconazole in the sensitive isolate (−50.9646 kcal/mol) was significantly less than that in the CYP51^S94A,N406S,H793R^ (−43.1264 kcal/mol) and CYP51^S94A,N406S,L750P,H793R^ (−37.5769 kcal/mol). In the interaction models of CYP51 with the sensitive isolate, a total of 11 amino acid residues had van der Waals forces with tebuconazole, such as PHE239, ILE498, etc. The TYR177, TYR231, TYR245, PHE345, PHE350, and ILE494 of CYP51 formed seven hydrophobic bindings of Pi-Alkyl with it. In the interaction models of CYP51^S94A,N406S,H793R^, there were eight amino acid residues having van der Waals forces with tebuconazole, TYR231, LEU234, TYR245, ALA425, ILE494, and ILE497, which produced seven hydrophobic bindings of Pi-Alkyl or Alkyl. More amino acid residues (14) had van der Waals forces with tebuconazole in the interaction models of CYP51^S94A,N406S,L750P,H793R^, but only HIS640, ALA644, TYR231, and ILE494 generated five hydrophobic bindings of Pi-Alkyl or Alkyl, and its PHE345 formed a Pi-Pi T-shaped interaction.

## 4. Discussion

Fungicide applications have been commonly used for the control of *R. solani* in China. However, the irrational and frequent usage of fungicide has caused a more serious problem: the proliferation of resistance genes [29]. There have been many reports about the resistance to different types of fungicides in *R. solani*, such as QoI fungicides [4]; however, little is known about the resistance to SDHI and DMI fungicides [9]. Our results showed that the EC50 values of the sensitive isolates were 0.03–0.05 μg/mL, consistent with the findings of Chen et al. (2012) [7], and the resistance levels of most resistant isolates were less than 5-fold, in addition to MSRS-2-7. At the same time, we also found that almost all the screened tebuconazole-resistant isolates reached the moderate level of resistance, especially LZHJ-1-8 isolate, which was not only highly resistant to tebuconazole, but also highly resistant to epoxiconazole. Resistance to DMI fungicide in many plant pathogens is of a quantitative nature characterized by slow shifts in sensitivity toward resistance [30].

Tebuconazole and epoxiconazole belong to DMI fungicides, which have been proven to bind with the heme part of CYP51 and inhibit demethylation of 24-methylenedihydro-lanosterol, a precursor of the cell membrane component ergosterol [31,32]. These are able to disrupt the cell membranes, causing an increase in relative electric conductivity [33]. Our results found that the relative electric conductivity of both tebuconazole-resistant and -sensitive isolates showed a significant increase tendency after tebuconazole treatment; meanwhile, we also found that although the tebuconazole-resistant isolates had an increased ability to tolerate tebuconazole and high osmotic pressure, their growth rate and relative electric conductivity with low sugar or tebuconazole were inferior to those of the sensitive strains, showing a fitness cost. Shao et al. (2015) [34] found that the laboratory-induced fluazinam-resistant mutants of *B. cinerea* were more sensitive to the osmotic stress than their fluazinam-sensitive parental isolates. Karaoglanidis et al. (2011) [35] found that the resistance to tebuconazole isolates had a significant adverse effect on the mycelial growth rate and pathogenicity.

We speculated that the resistance to DMI fungicides and its fitness cost is due to the pleiotropy caused by the mutation of the target CYP51 [36]. Some CYP51 mutations reduce the affinity between the target protein and pesticide, which is one of the important factors leading to the production of resistance [37], and may also affect fungal sterol synthesis and, thus, its fitness [38]. Our CYP51 mutation detection results showed that all the screened tebuconazole-resistant isolates had site mutations, while CYP51^S94A,N406S,H793R^ mutations were the main resistant population types. Pereira et al. [39] found that the resistance to tebuconazole had a significant linear correlation with the G461S mutation frequency. Stammler et.al (2012) [40] indicated that some mutations in CYP51 (e.g., mutations A379G, I381V), were known to be an adaptive response to DMIs that had already disappeared in Northern Europe. Other mutations in the CYP51 gene, e.g., G143A in V136A, A379G, I381V, and mutations or deletions at the amino acid positions 459–462 of CYP51 in *M. graminicola* [41], Y134F in *Puccinia triticina* [42], *Z. tritici* [43], Y137H in *Fusarium graminearum* [44] etc., were proven to be related with DMI fungicide resistance. The K motif and heme-binding motif constituted the conserved domains characteristic of P450 proteins [45]. Although S94A, N406S, L750P, and H793R were not located in these motifs, six substrate recognition sites (SRS1-6) have been identified to contain the amino acid residue S, N, or L [46]. Our results also showed that the binding energy of CYP51 and tebuconazole in the sensitive isolate was significantly less than that of CYP51^S94A,N406S,H793R^ and CYP51^S94A,N406S,L750P,H793R^. Therefore, the point mutation on the target gene CYP51 of tebuconazole was associated with the resistance of rice bacterial strain to tebuconazole. In the next step, we will use site-directed mutagenesis to edit CYP51 of *R. solani* to verify the relationship between these mutations and resistance, and to explore the mechanism by which mutations lead to changes in the fitness cost and resistance.

## 5. Conclusions

The resistance frequency results of 122 *R. solani* isolates by MIC showed that the resistance frequency for several fungicides was different, and the resistance frequency of tebuconazole reached 47.54%. Furthermore, a moderate resistance to thifluzamide was identified in isolate MSRS-2-7, whereas most isolates were sensitive to hexaconazole or epoxiconazole. In addition, a highly tebuconazole-resistant isolate LZHJ-1-8 displayed a moderate resistance to epoxiconazole, and the screened tebuconazole-resistant isolates showed a moderate or high resistance to tebuconazole. The tebuconazole-resistant isolates showed higher fitness costs with a lower growth rate and higher relative electric conductivity, demonstrating an increased ability to tolerate tebuconazole and high osmotic pressure. CYP51 mutation results showed that the tebuconazole-resistant isolates retained S94A, N406S, and H793R mutations, while LZHJ-1-8 possessed another mutation, L750P, which supported the lowest binding energy with tebuconazole. These results suggest that S94A, N406S, L750P, and H793R mutations of CYP51 may be linked to the resistance to tebuconazole.

## Figures and Tables

**Table 1:** The frequency of resistance to *R. solani* isolates in Sichuan Province.

Sampling Sites	Thifluzamide			Hexaconazole			Tebuconazole			Epoxiconazole		
	R	S	Res-Frequency	R	S	Res-Frequency	R	S	Res-Frequency	R	S	Res-Frequency
CDCZ	11	8	57.89%	18	1	94.74%	11	8	57.89%	13	6	68.42%
CDDY	2	1	66.67%	3	0	100.00%	1	2	33.33%	1	2	33.33%
CDPX	3	5	37.50%	2	6	25.00%	0	8	0.00%	3	5	37.50%
MSRS	30	10	75.00%	8	32	20.00%	7	33	17.50%	7	33	17.50%
ZGRX	28	11	71.79%	18	21	46.15%	28	11	71.79%	14	25	35.90%
LZHJ	4	4	50.00%	6	2	75.00%	7	1	87.50%	5	3	20.83%
NJLC	2	3	40.00%	0	5	0.00%	4	1	80.00%	1	4	20.00%
Total	80	42	65.57%	55	67	45.08%	58	64	47.54%	44	78	36.07%

R: Resistant isolates; S: Susceptible isolates; Res-frequency: resistance frequency.

**Table 2:** Determination of sensitivity baseline of *R. solani* isolates to four tested fungicides.

Test Fungicides	No. of Isolates	Regression Equation	EC_50_ (μg/mL)	95% Confidence Interval	Correlation Index
Thifluzamide	CDPX-1-7	Y = 5.8483 + 0.6012 X	0.04	(0.02–0.09)	0.9656
	ZGRX-2-13	Y = 6.0421 + 0.7504 X	0.04	(0.02–0.07)	0.9877
	MSRS-2-17	Y = 5.8319 + 0.6303 X	0.05	(0.03–0.09)	0.9625
	CDPX-1-3	Y = 5.9502 + 0.6050 X	0.03	(0.01–0.07)	0.9583
	ZGRX-2-19	Y = 6.2788 + 0.8061 X	0.05	(0.01–0.06)	0.9665
	CDPX-1-7	Y = 6.4815 + 0.9369 X	0.03	(0.01–0.05)	0.9715
	CDDY1-2	Y = 6.2350 + 0.9628 X	0.05	(0.03–0.08)	0.9844
	MSRS-2-3	Y = 5.8892 + 0.6085 X	0.04	(0.02–0.09)	0.9825
	ZGRX-3-1	Y = 5.8052 + 0.6085 X	0.05	(0.02–0.09)	0.9706
	ZGRX-1-3	Y = 6.5188 + 1.0734 X	0.04	(0.03–0.06)	0.9370
Hexaconazole	ZGRX-3-1	Y = 5.9980 + 0.8225 X	0.06	(0.04–0.09)	0.9998
	ZGRX-2-3	Y = 6.7999 + 1.4116 X	0.05	(0.04–0.07)	0.9882
	MSRS-3-13	Y = 6.6624 + 1.0217 X	0.02	(0.01–0.04)	0.9876
	MSRS-2-12	Y = 6.7086 + 1.1962 X	0.04	(0.03–0.06)	0.9988
	MSRS-3-6	Y = 6.6118 + 1.1475 X	0.04	(0.03–0.06)	0.9977
	MSRS-3-16	Y = 6.7126 + 1.3524 X	0.05	(0.04–0.08)	0.9517
	MSRS-2-6	Y = 6.9664 + 1.7276 X	0.07	(0.05–0.10)	0.9672
	MSRS-3-17	Y = 6.7536 + 1.4386 X	0.06	(0.04–0.09)	0.9947
	ZGRX-3-2	Y = 5.9080 + 0.7256 X	0.06	(0.03–0.12)	0.9886
	ZGRX-2-19	Y = 6.1238 + 0.9151 X	0.06	(0.03–0.11)	0.9926
Tebuconazole	CDCZ-1-15	Y = 5.5328 + 0.6969 X	0.17	(0.12–0.26)	0.9912
	ZGRX-2-3	Y = 5.6929 + 0.7746 X	0.13	(0.09–0.19)	0.9878
	CDCZ-1-10	Y = 5.5518 + 0.6825 X	0.16	(0.10–0.24)	0.9852
	MSRS-3-16	Y = 7.2183 + 1.3364 X	0.02	(0.01–0.06)	0.9739
	MSRS-3-10	Y = 7.2672 + 1.8764 X	0.06	(0.05–0.08)	0.9963
	CDPX-1-7	Y = 6.5625 + 1.3526 X	0.07	(0.05–0.10)	0.9850
	MSRS-3-13	Y = 7.3102 + 1.8224 X	0.05	(0.04–0.08)	0.9684
	CDPX-1-6	Y = 6.4646 + 1.4190 X	0.09	(0.07–0.12)	0.9947
	CDCZ-1-6	Y = 5.9860 + 0.7552 X	0.05	(0.03–0.09)	0.9683
	MSRS-2-13	Y = 5.5530 + 0.8550 X	0.23	(0.16–0.31)	0.9893
Epoxiconazole	MSRS-2-13	Y = 7.0486 + 1.3978 X	0.03	(0.02–0.05)	0.9839
	MSRS-2-12	Y = 6.0911 + 0.8262 X	0.05	(0.03–0.08)	0.9725
	MSRS-2-6	Y = 5.9836 + 0.8701 X	0.07	(0.05–0.11)	0.9132
	MSRS-2-17	Y = 5.9911 + 1.1652 X	0.14	(0.10–0.19)	0.9826
	MSRS-3-10	Y = 6.0264 + 0.9587 X	0.08	(0.05–0.13)	0.9878
	MSRS-3-13	Y = 6.1238 + 0.8946 X	0.06	(0.04–0.08)	0.9768
	ZGRX-1-3	Y = 6.0133 + 1.1836 X	0.14	(0.11–0.18)	0.9971
	ZGRX-1-1	Y = 5.9971 + 1.2023 X	0.15	(0.09–0.24)	0.9951
	CDCZ-1-9	Y = 5.8796 + 1.0372 X	0.14	(0.09–0.23)	0.9829
	CDDY1-2	Y = 5.7995 + 0.9206 X	0.14	(0.08–0.24)	0.9826

**Table 3:** Determination of resistance levels of *R. solani* isolates to four tested fungicides.

Test Fungicides	No. of Isolates	Regression Equation	EC_50_ (μg/mL)	95% Confidence Interval	Correlation Index	Resistance Fold
Thifluzamide	MSRS-2-7	Y = 5.1309 + 0.8174 X	0.69	(0.30–1.59)	0.9825	16.43
	CDCZ-1-7	Y = 5.5432 + 1.0143 X	0.29	(0.16–0.53)	0.9783	6.90
	CDPX-1-3	Y = 5.6393 + 0.7784 X	0.15	(0.09–0.25)	0.9493	3.57
	ZGRX-2-1	Y = 6.1624 + 1.1256 X	0.09	(0.07–0.12)	0.9557	2.14
	MSRS-3-6	Y = 5.6066 + 0.5882 X	0.09	(0.05–0.18)	0.8505	2.14
	MSRS-3-13	Y = 6.7033 + 1.5839 X	0.08	(0.05–0.13)	0.9441	1.90
	ZGRX-2-20	Y = 6.3143 + 1.1751 X	0.08	(0.05–0.12)	0.9937	1.90
	ZGRX-2-4	Y = 5.7924 + 0.6502 X	0.06	(0.03–0.11)	0.9330	1.43
	ZGRX-3-4	Y = 5.1309 + 0.8714 X	0.07	(0.30–1.59)	0.9825	1.67
	LZHJ-1-3	Y = 7.9279 + 2.4365 X	0.06	(0.05–0.08)	0.8846	1.43
Hexaconazole	CDPX-1-5	Y = 5.9542 + 1.2631 X	0.18	(0.11–0.28)	0.9333	4.50
	MSRS-2-8	Y = 6.0407 + 1.1371 X	0.12	(0.08–0.18)	0.9880	3.00
	CDCZ-1-16	Y = 6.2837 + 1.7042 X	0.18	(0.14–0.22)	0.9693	4.50
	CDPX-1-3	Y = 6.0707 + 1.2022 X	0.13	(0.09–0.18)	0.9847	3.25
	MSRS-2-20	Y = 5.7696 + 0.9057 X	0.14	(0.09–0.22)	0.9877	3.50
	CDCZ-1-19	Y = 6.2636 + 1.6420 X	0.17	(0.13–0.22)	0.9783	4.25
	ZGRX-1-1	Y = 6.2531 + 1.5260 X	0.15	(0.11–0.21)	0.9709	3.75
	CDCZ-1-18	Y = 6.5664 + 1.8577 X	0.14	(0.11–0.19)	0.9893	3.50
	CDPX-1-4	Y = 5.8777 + 0.8436 X	0.09	(0.05–0.16)	0.9277	2.25
	MSRS-2-11	Y = 5.8841 + 0.8176 X	0.08	(0.04–0.16)	0.9984	2.00
Tebuconazole	LZHJ-1-4	Y = 5.1587 + 1.1307 X	0.72	(0.54–0.97)	0.9715	14.12
	ZGRX-3-4	Y = 4.8774 + 0.9424 X	1.35	(0.96–1.90)	0.9563	26.47
	MSRS-1-3	Y = 4.5709 + 1.0107 X	2.66	(1.64–4.30)	0.9679	52.16
	LZHJ-1-2	Y = 5.2758 + 1.1849 X	0.59	(0.43–0.80)	0.9887	11.57
	MSRS-2-11	Y = 4.9065 + 1.1902 X	1.20	(0.89–1.61)	0.9717	23.53
	CDCZ-1-19	Y = 5.2303 + 0.9686 X	0.58	(0.38–0.87)	0.9811	11.37
	CDCZ-1-9	Y = 4.9063 + 0.9036 X	1.27	(0.88–1.83)	0.9795	24.90
	CDCZ-1-7	Y = 5.1560 + 0.5903 X	0.54	(0.28–1.05)	0.9798	10.59
	MSRS-2-17	Y = 4.7848 + 1.3253 X	1.45	(1.09–1.94)	0.9878	28.43
	LZHJ-1-8	Y = 4.2535 + 1.5181 X	3.10	(1.99–4.84)	0.9996	60.78
Epoxiconazole	MSRS-2-11	Y = 6.1163 + 1.7336 X	0.23	(0.15–0.34)	0.9971	2.30
	MSRS-2-17	Y = 5.9897 + 1.6367 X	0.25	(0.19–0.33)	0.9982	2.50
	ZGRX-2-20	Y = 5.6710 + 1.0176 X	0.22	(0.14–0.33)	0.9866	2.20
	MSRS-2-8	Y = 5.6199 + 1.4031 X	0.36	(0.28–0.46)	0.9839	3.60
	CDPX-1-3	Y = 5.8153 + 1.4081 X	0.26	(0.20–0.34)	0.9954	2.60
	LZHJ-1-3	Y = 5.9860 + 1.5160 X	0.22	(0.17–0.30)	0.9821	2.20
	ZGRX-3-4	Y = 5.3441 + 0.8510 X	0.39	(0.22–0.72)	0.9431	3.90
	MSRS-2-7	Y = 5.5624 + 0.9933 X	0.27	(0.17–0.43)	0.9923	2.70
	LZHJ-1-8	Y = 4.4986 + 0.9143 X	3.54	(1.33–9.38)	0.9804	35.40
	MSRS-3-16	Y = 5.6143 + 0.8581 X	0.19	(0.12–0.30)	0.9619	1.90

**Table 4:** Statistics of point mutation information in CYP51 gene.

Isolate Type	Code	Resistant Type	Mutational Type
Susceptible isolate	MSRS-3-16	-	-
	ZGRX-2-3	-	-
	CDCZ-1-15	-	-
	CDCZ-1-7	-	-
Resistant isolate	ZGRX-3-4	MR	S94A, N406S, H793R
	MSRS-2-17	MR	S94A, N406S, H793R
	MSRS-1-3	HR	S94A, N406S, H793R
	LZHJ-1-8	HR	S94A, N406S, L750P, H793R

## Data Availability

Not applicable.

## Supplementary Materials

The following supporting information can be downloaded at: https://www.mdpi.com/article/10.3390/cimb44100330/s1, Figure S1: Meme diagram of sterol sterol 14α-demethylase from different species; Table S1: The source, quantity and number of all *R. solani* isolates; Table S2: Primers for CYP51 cloning.
