# Supplementary material for: Status of Fungicide Resistance and Physiological Characterization of Tebuconazole Resistance in Rhizocotonia solani in Sichuan Province, China
Source: Curr Issues Mol Biol. 2022 Oct 13;44(10):4859–76. doi: 10.3390/cimb44100330 (PMC9600323; doi:10.3390/cimb44100330)
Supplement: Supplementary file 1 [file cimb-44-00330-s001.zip › cimb-1932558-supplementary.pdf]

Table S1. The source, quantity and number of all *R. solani* isolates

Sampling area	Serial number	Number	Sampling time	Separation time	Latitude and longitude
CDCZ	CDCZ-1-1~19	19	2018.08.22	2018.08.27	104º08′ 30º66′
CDDY	CDDY-1-1~3	3	2018.08.21	2018.08.24	103º50′ 30º52′
CDPX	CDPX-1-1~8	8	2018.08.22	2018.08.26	103º99′ 30º82′
MSRS	MSRS-1-1~3	3	2018.08.20	2018.08.25	104º18′ 29º82′
MSRS	MSRS-2-1~20	20	2018.08.20	2018.08.25	104º15′ 29º84′
MSRS	MSRS-3-1~17	17	2018.08.20	2018.08.25	104º47′ 29º86′
ZGRX	ZGRX-1-1~9	9	2018.08.08	2018.08.12	104º19′ 29º58′
ZGRX	ZGRX-2-1~24	24	2018.08.08	2018.08.12	104º26′ 29º50′
ZGRX	ZGRX-3-1~6	6	2018.08.08	2018.08.12	104º24′ 29º44′
LZHJ	LZHJ-1-1~8	8	2019.08.16	2019.08.19	105º84′ 28º82′
NJLC	NJLC-1-1~5	5	2018.08.10	2018.08.13	105º17′ 29º35′

Table S2. Primers for *CYP51* c*loning*

Primer name	Primer sequence	Fragment length
CYP51-F1	ATCGAGTATTCAAGGGTTATAG	1180bp
CYP51-R1	TTATCATTTCCGTCACACACTTATT	
CYP51-F2	CGATTAAAGAGAAGATGACGGTAGA	1062 bp
CYP51-R2	TTAAGCGAACAAGCAACTGATCC	
CYP51-F3	TGGTTGATACGGGCTCTCAGTTC	1311 bp
CYP51-R3	TGAGGAAGCCTATACGGTCAGTTTA	
CYP51-F4	GTAAGTCCGAATTTGACAAATTTCT	1021 bp
CYP51-R4	CTTGTGATATCCTCTGGCTTTGT	

Motifs

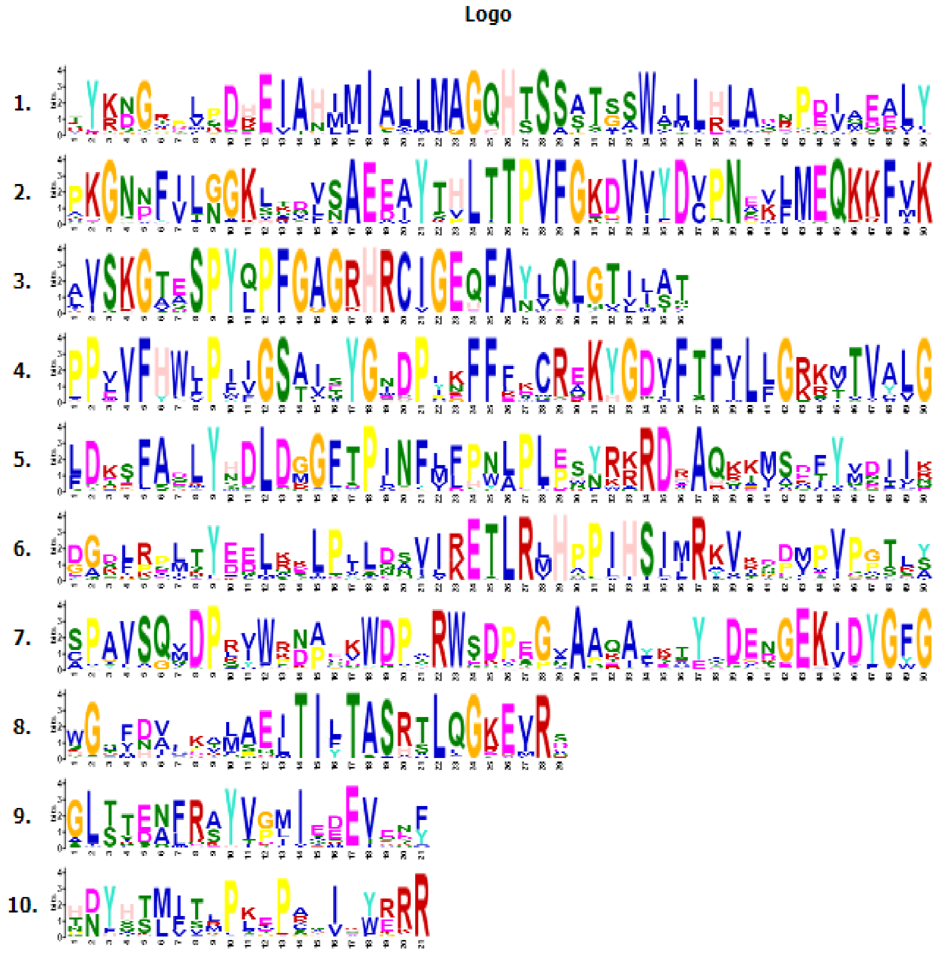

Figure S1. Meme diagram of sterol sterol 14α-demethylase from different species.
Note: MEME1 to MEME10 represent motif1 to motif10, which are structurally conserved domains retrieved through meme, respectively.
